# Targeting NRF2 With Isoeugenol: A Promising Small Molecule for Neurodegenerative, Metabolic, and Chronic Inflammatory Disorders

**DOI:** 10.1155/omcl/7695056

**Published:** 2025-12-12

**Authors:** Ana Silva, Sónia Silva, Beatriz Rodrigues, Gonçalo Simões, Inês Dinis, Mafalda Freitas, Rosa Resende, Joana Bicker, Ana Fortuna, Maria M. Silva, Armanda E. Santos, Sónia A. Pinho, Bruno Neves, Cláudia Fragão Pereira, Maria Teresa Cruz

**Affiliations:** ^1^ Center for Neuroscience and Cell Biology, University of Coimbra, Coimbra, 3000-548, Portugal, uc.pt; ^2^ Faculty of Pharmacy, Center for Neuroscience and Cell Biology, University of Coimbra, Coimbra, 3000-548, Portugal, uc.pt; ^3^ CIBB, Centre for Innovative Biomedicine and Biotechnology, University of Coimbra, Coimbra, 3000-548, Portugal, uc.pt; ^4^ CAC-CHUC, Clinical Academy Center of Coimbra Hospital and University Center, Coimbra, Portugal; ^5^ CEMMPRE—Centre for Mechanical Engineering, Materials and Processes, Coimbra, Portugal; ^6^ CIBIT/ICNAS - Coimbra Institute for Biomedical Imaging and Translational Research, University of Coimbra, Azinhaga de Santa Comba, Coimbra, 3000-548, Portugal, uc.pt; ^7^ iBiMED, Department of Medical Sciences and Institute of Biomedicine, University of Aveiro, Aveiro, 3810-193, Portugal, ua.pt; ^8^ Faculty of Medicine, University of Coimbra, Coimbra, 3000-548, Portugal, uc.pt

**Keywords:** Alzheimer’s disease, diabetes, inflammation, isoeugenol, NRF2, skin allergen

## Abstract

Oxidative stress, driven by an imbalance between oxidants and antioxidants, disrupts redox homeostasis and contributes to the development of chronic diseases, including cancer, diabetes, neurodegenerative disorders, and aging. The NRF2‐KEAP1 pathway is a pivotal cellular defense mechanism against oxidative stress, regulating the transcription of cytoprotective genes. Pharmacological NRF2 activation has emerged as a promising strategy to mitigate oxidative stress‐related pathologies; however, challenges regarding target specificity, pharmacodynamics, efficacy, and safety remain unresolved. Isoeugenol, a phenylpropanoid found in essential oils, has traditionally been recognized as a skin allergen but is now gaining attention for its potential as an NRF2 activator. Emerging evidence suggests that isoeugenol exerts antioxidant, anti‐inflammatory, and neuroprotective effects and modulates metabolic disorders such as diabetes mellitus. Despite its therapeutic potential, the direct correlation between isoeugenol’s effects and NRF2 activation remains underexplored. Existing studies indicate that isoeugenol may activate NRF2 through multiple mechanisms, including covalent modification of KEAP1 cysteine residues, increased AKT activation and GSK3β inactivation, and glutathione depletion leading to reactive oxygen species (ROS) generation. Understanding these activation pathways is critical for leveraging isoeugenol as a therapeutic agent. This review provides a comprehensive analysis of isoeugenol’s role in modulating NRF2 activity and its implications for treating oxidative stress‐driven diseases. By integrating current findings, this review highlights new insights into the therapeutic potential of isoeugenol in translational medicine. We propose future research directions to optimize its application in clinical settings, paving the way for more targeted and effective NRF2‐based interventions in chronic disease management.

## 1. Introduction

### 1.1. The KEAP1–NRF2 Pathway

Oxidative stress plays a fundamental role in the initiation and progression of various chronic diseases, including diabetes, auto‐immune, cardiovascular, and neurodegenerative diseases. In a physiological context, redox homeostasis is maintained through the balance between the production of reactive oxygen species (ROS), such as the superoxide radical, the hydrogen peroxide, and the hydroxyl radical, and the concentrations of antioxidants, including glutathione and the antioxidant enzymes glutamate‐cysteine ligase, NADPH quinone oxidoreductase 1 (NQO1) and heme oxygenase‐1 (HO‐1), among others [1, 2]. Oxidative stress occurs when there is a disturbance in this equilibrium, due to compromised defense mechanisms and/or increased generation of free radicals. This harmful condition damages nucleic acids, proteins, lipids, and membranes, resulting in tissue injury and cell death [1, 2]. The cytoprotective transcription factor nuclear factor erythroid 2‐related factor 2 (NRF2) is part of a complex regulatory network that responds to environmental stimuli. The evolutionary development of the NRF2‐Kelch‐like ECH‐associated protein 1 (KEAP1) signaling pathway has been crucial for sustaining life on Earth, ensuring the regulation of antioxidant gene expression, inflammation, detoxification, and protein homeostasis. Indeed, as the atmospheric oxygen levels increased, mammals needed mechanisms to protect themselves from the metabolic toxicity caused by ROS production. Examples from the animal kingdom suggest that enhanced NRF2 expression and superior antioxidant defense mechanisms have evolved to protect animals under extreme environmental conditions, such as deep‐sea diving, hibernation, and habitual hypoxia [3]. Therefore, pharmacological activation of NRF2 is a promising therapeutic approach for several chronic diseases that are underlined by oxidative stress and inflammation, such as neurodegenerative, cardiovascular, and metabolic diseases [4].

NRF2 is a basic leucine zipper transcription factor (bZIP) characterized by six extensively conserved domains known as NRF2‐ECH homology (Neh) domains. The distinct functional roles of each Neh domain are precisely delineated. The Neh2 domain contains an N‐terminal region allowing the binding to KEAP1, which negatively controls NRF2 activity [2, 4, 5]. Indeed, NRF2 activity is tightly regulated by two main mechanisms that control NRF2 stability in the cytoplasm. Under physiological conditions, NRF2 binds to KEAP1, a ubiquitin E3 ligase adapter comprising several redox‐ and electrophilic‐sensitive residues of cysteine, which presents NRF2 to a Cullin3/Rbx1‐dependent E3 ubiquitin ligase complex that promotes NRF2 ubiquitination and further degradation in the proteasome (Figure 1Aa). The other mechanism involves the phosphorylation of specific NRF2 serine residues by the glycogen synthase kinase‐3β (GSK‐3β), leading to substrate recognition by β‐transducin repeat containing E3 ubiquitin protein ligase (β‐TrCP, also an E3 ligase adapter), which signals NRF2 for ubiquitination mediated by the Cullin‐1/Rbx1 complex, resulting in its proteasomal degradation. GSK‐3β is inhibited when phosphorylated at Ser9 by Ser/Thr protein kinases, such as AKT. Hence, it has been suggested that the activation of AKT and consequent inactivation of GSK‐3β might upregulate NRF2 [6–8] (Figure 1A A1).

Figure 1KEAP1–NRF2 pathway. (A A1). Under physiological conditions, KEAP1 mediates the ubiquitination of NRF2 through its interaction with the Cullin3/Rbx1 dependent E3 ubiquitin ligase complex, leading to the degradation of NRF2. (A2). GSK‐3β phosphorylates NRF2, developing a recognition region for β‐TrCP, which triggers the Cullin1/Rbx1 complex, leading to NRF2 ubiquitination and its degradation in the proteasome. (B) Several pharmacological NRF2 activators are electrophilic molecules able to inhibit the KEAP1 protein by modification and oxidation of specific cysteine residues, thus releasing NRF2, which translocates to the nucleus and initiates the transcription of 250 genes involved in redox metabolism, proteostasis, and inflammation.

**Figure:**
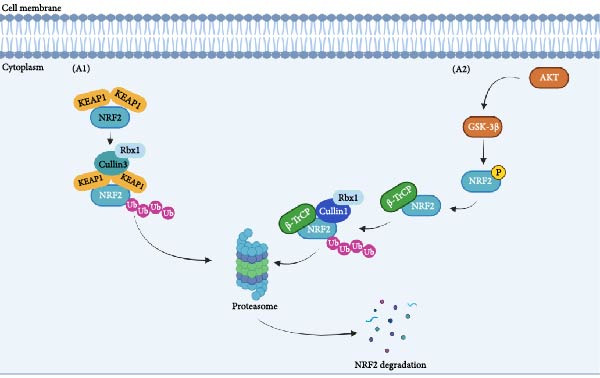
(A)

**Figure:**
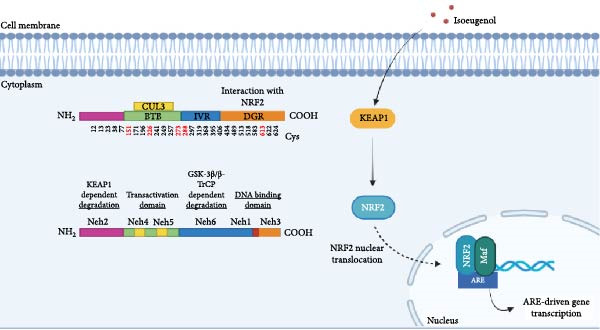
(B)

Most pharmacological NRF2 activators are electrophilic molecules that covalently modify cysteine residues present in the thiol‐rich KEAP1 protein by oxidation or alkylation [9–11]. Indeed, KEAP1 is a 624‐amino‐acid protein with five domains and 27 cysteine residues. Some of these cysteine residues are redox sensitive and their oxidation by electrophiles causes inactivation of KEAP1. Interestingly, NRF2‐activating electrophiles have different specificities for the cysteine residues of KEAP1, termed the cysteine code, although they all inactivate KEAP1 and therefore activate NRF2. Many cysteines of KEAP1 are modified by different electrophiles and cysteines Cys‐151, Cys‐273, and Cys‐288 appear to be the most susceptible to electrophile reaction [12–18]. Other sensitive cysteines include Cys‐226, Cys‐434, and Cys‐613. This “cysteine code” regulates KEAP1 activity when a protective response mediated by NRF2 is required. The covalent modification of cysteine residues in the KEAP1 protein prevents it from targeting NRF2 for ubiquitination. As a result, NRF2 migrates to the nucleus, where it binds to small musculoaponeurotic fibrosarcoma (Maf) proteins and antioxidant response element (ARE) sequences, initiating the transcription of 250 genes involved in a multitude of cellular functions that include redox metabolism, proteostasis, and inflammation [2, 4–6, 19] (Figure 1B).

Our group has been working with low molecular skin allergens in the last 20 years. and, interestingly, NRF2 activation is a key event triggered by these small molecules. This regulatory pathway is indeed the most common regulatory pathway activated by sensitizers at the gene expression level, and the underlying event in keratinocytes has become formalized as a Key Event in the Organisation for Economic Co‐operation and Development (OECD) Adverse Outcome Pathway for skin sensitization. These studies led to the development of the KeratinoSens assay, which measures the activation of NRF2 in keratinocytes and became the first cell‐based in vitro test for skin sensitization hazard to be endorsed by a statement from the European Union Reference Laboratory for Alternatives to Animal Testing (EURL ECVAM) and an OECD Test Guideline [20].

The activation of NRF2 by skin allergens is well explained by their direct reactivity towards key cysteine residues of KEAP1 protein, causing its dissociation and subsequently promoting the translocation of NRF2 to the nucleus [21]. Interestingly, dimethyl fumarate (DMF), a known skin allergen [22], was approved by the FDA and EMA for the treatment of multiple sclerosis in 2013 [23], due to its NRF2‐activating properties. Indeed, DMF inactivates KEAP1 via oxidation of cysteine 151, causing nuclear translocation of NRF2, NRF2 target gene expression and repression of proinflammatory cytokine. Beyond its Nrf2‐dependent effects, DMF also influences other pathways, including the inhibition of NF‐κB, modulation of immune responses, regulation of cell fate, and potential effects on oxidative stress and mitochondrial function, highlighting its multifaceted mechanisms of action [24]. Besides DMF, omaveloxolone has more recently been approved for the treatment of Friedrich ataxia as an electrophilic Nrf2 activator [25], underscoring the growing therapeutic potential of Nrf2 activation in neurodegenerative diseases and highlighting the broader relevance of Nrf2 modulation beyond multiple sclerosis. This indicates that skin allergens, far from being solely associated with toxic effects, can initiate beneficial protective pathways, presenting a unique potential often overlooked in drug discovery programs. In this review article, we will disclose the impact of the skin allergen isoeugenol, an activator of NRF2, across various disease contexts characterized by oxidative stress and inflammation, including neurodegenerative, inflammatory, and metabolic disorders.

## 2. The Skin Allergen Isoeugenol

**Figure 2:**
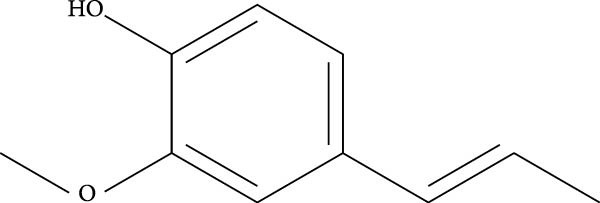
Chemical structure of isoeugenol.

Isoeugenol (2‐methoxy‐4‐(1‐propenyl)phenol) is a phenylpropanoid extracted from the essential oils of various spices, including clove, nutmeg, and cinnamon and well acknowledged to possess antioxidant and thus cytoprotective activities (Figure 2). This small lipophilic molecule is widely used in the food and cosmetics industries, despite being a skin allergen with electrophilic properties, requiring the cosmetics and fragrance industries to disclose its presence in manufactured products [26, 27].

**Figure 3:**
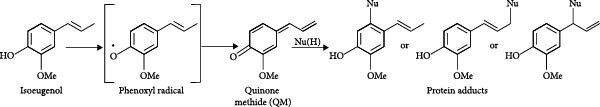
Oxidation of isoeugenol into the electrophilic intermediate quinone methide (QM) and subsequent formation of Michael adducts with proteins, proposed by Bertrand 1997. Nu(H), nucleophile.

Being a phenolic compound, isoeugenol exhibits expectable antioxidant activity, being able to trap reactive oxygen radicals by the donation of the phenolic hydrogen atom, thus forming a phenoxyl radical [28]. At higher concentrations, isoeugenol has been shown to enhance ROS production leading to depletion of glutathione [29]. Glutathione depletion is a key factor in the pro‐oxidant effects of several nature‐derived electrophiles, which, along with their indiscriminate reactivity, often contribute to their side effects. However, it is important to acknowledge that, in some contexts, this pro‐oxidant activity can also lead to beneficial outcomes, such as the activation of NRF2 and stress response pathways that enhance cellular defense mechanisms. The activation of Nrf2 can counterbalance the reactive oxygen radicals generated, mitigating their harmful effects, while simultaneously triggering other protective events, such as anti‐inflammatory responses, inhibition of ferroptosis, and enhancement of autophagy, all of which contribute to cellular survival and tissue homeostasis. Furthermore, isoeugenol is prone to radical oxidation [30] (whether enzymatic or non‐enzymatic) leading to the generation of the reactive specie quinone methide (QM). Isoeugenol is not a direct Michael acceptor and is devoid of a structural alert feature but can be considered a pre‐hapten due to the presence of a pre‐Michael acceptor domain. The oxidation of isoeugenol in vivo has been subjected to several mechanistic studies [31]. It was proposed that the QM derivative would act as a Michael acceptor and react with nucleophilic residues of proteins in vivo (Figure 3).

**Figure 4:**
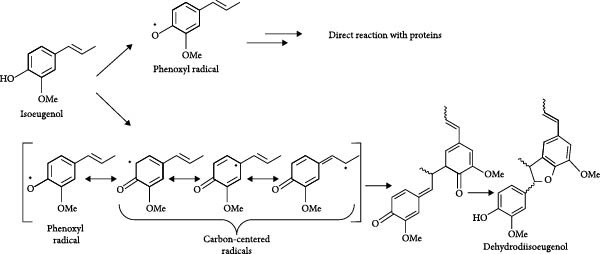
Formation pathway of the phenoxyl radical and of carbon‐centered radicals, followed by dimerization. Adapted from Port‐Lougarre [28].

Recently, it has been shown that the phenoxyl radical formed upon donation of the hydrogen atom to oxidative reactive species, can itself react with proteins before conversion into QM [28]. Furthermore, under light exposure, the radical can delocalize to carbon positions in the isoeugenol structure, leading to the formation of isoeugenol dimers (dehydrodiisoeugenol, as a mixture of diastereomers) [28] (Figure 4). These dimers have been described as moderate skin sensitizers [32]. Therefore, isoeugenol may undergo either biological or physical activation into reactive derivatives, exhibiting a prohapten and prehapten nature.

**Figure 5:**
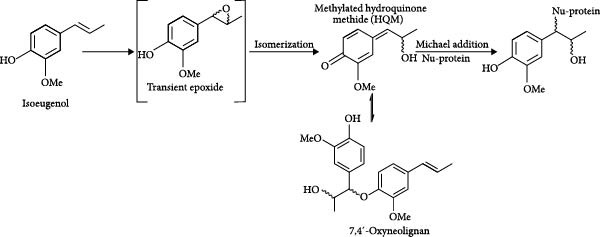
Formation pathway of the electrophilic intermediate: methylated hydroxyquinone methide (HQM) and of the dimer 7,4ʹ‐oxyneolignan. Adapted from Ahn et al. [26] and Ahn et al. [31].

On the other hand, it has been proposed that epoxidation of isoeugenol leads to the formation of a hydroxy QM (HQM) [31]. This derivative can undergo dimerization into diastereomeric 7,4ʹ‐oxyneolignans, which can react with thiol groups present in cysteine residues [26], or act as a Michael acceptor and trigger covalent reactions when they come into contact with the amino and thiol groups of skin proteins (Figure 5). This interaction activates immune pathways that culminate in the development of skin sensitization. However, when administered via noncutaneous routes, electrophilic derivatives of isoeugenol may exert therapeutic effects by covalently binding to cysteine residues on the KEAP1 protein, thereby activating NRF2 without inducing dermatological toxicity. This mechanism parallels that of DMF, a Michael acceptor and skin allergen.

The mechanisms underlying Keap1 inhibition by isoeugenol need to be clarified, but we can anticipate that the relatively stable phenoxyl radical (Figure 4) may interact with Keap1, namely oxidizing the thiol groups of cysteine, forming disulfide linkages [33]. Furthermore, direct alkylation of Keap1 by the methylated HQM (Figure 4) or by the dimeric derivatives (Figures 4 and 5) after oxidative activation can also occur. On the other hand, noncovalent interactions may be established between the dimeric derivatives and Keap1, preventing protein–protein interactions with Nrf2, as extensively reviewed by Abed et al. [34].

Also, we previously demonstrated, using an in chemico assay developed by our team, that isoeugenol induces peptide depletion—specifically of glutathione—through the formation of adducts with cysteine, resulting in 100% depletion. This depletion leads to ROS generation, which not only acts as a well‐established activator of NRF2 but may also represent an additional mechanism through which isoeugenol triggers NRF2 activation [35].

Therefore, we advocate for an in‐depth exploration of low molecular weight skin allergens, and we considered isoeugenol as a pivotal starting point in this field, highlighting its potential to pave the way for new therapeutic approaches that leverage NRF2 activation to combat oxidative stress and inflammation. In the following sections, pharmacological activities attributable to isoeugenol will be discussed. Of note, some of the studies have not been directly linked to NRF2 activation by the authors but are likely influenced by this pathway. This exploration aims to highlight the broader therapeutic potential of isoeugenol, and similar compounds activators of NRF2, in mitigating oxidative stress and inflammation across various disease contexts.

## 3. Pharmacological Activities of Isoeugenol

### 3.1. Antioxidant Properties

The antioxidant activity of isoeugenol and its derivatives has been extensively reported in the literature [36]. It is a potent free radical scavenger that protects against cisplatin‐induced toxicity both in vitro and in vivo [37] and against DNA oxidative damage caused by hydroxyl radicals, in a molecular simulation model [38]. Additionally, it protects against metal‐mediated lipid peroxidation and isoeugenol antioxidant activity is attributed to its methoxyphenolic structure [39]. A study conducted by Atsumi et al. to comparatively assess the antioxidant and pro‐oxidant activities of isoeugenol and eugenol, another skin allergen and a structural isomer of isoeugenol, concluded that isoeugenol functions as an antioxidant and anti‐inflammatory agent at lower concentrations. Conversely, at higher concentrations, it exhibits pro‐oxidant characteristics, as evidenced by increased ROS production and a significant reduction in glutathione levels [29]. Accordingly, isoeugenol is capable of modulating the activity of glutathione, responsible for protection against oxidative damage. The thiol groups of cysteine in this endogenous antioxidant are responsible for its activity. In this sense, isoeugenol, being an electrophilic molecule, can conjugate with the thiol groups, impacting the beneficial activity of glutathione within the cells. This depletion of functional glutathione levels promotes the expression of HO‐1, which, in turn, contributes to an anti‐inflammatory and immunosuppressive response [2, 19]. Focusing on the role of HO‐1, it can convert heme, a pro‐oxidant that is formed in response to periods of metabolic stress, into three bioactive substances with antioxidant and anti‐inflammatory properties, which are free iron, carbon monoxide, and biliverdin. Afterwards, through biliverdin reductase, biliverdin is converted into bilirubin, which also has an antioxidant role [19]. Prasad et al. [40] evaluated the propensity of eugenol and isoeugenol to ameliorate the acrylamide‐induced neurotoxicity in *Drosophila melanogaster*. Eugenol and isoeugenol enriched diet offered marked protection against acrylamide‐induced mortality, locomotor dysfunctions, and oxidative stress. Furthermore, both molecules maintained the activity of acetylcholinesterase enzyme and dopamine levels in the head region. Collectively, these findings demonstrate that acrylamide‐induced neurotoxicity in Drosophila may be mediated through oxidative stress mechanisms and the potential of isoeugenol and eugenol to abrogate the condition. Additionally, these beneficial effects were later confirmed in acrylamide‐induced neuropathy in rats. Isoeugenol has shown detoxifying properties and the ability to eliminate ROS, NO, malondialdehyde, and hydrogen peroxide in the sciatic nerve and brain regions highlighting its possible therapeutic usage as an adjuvant in the management of neuropathy in humans [41]. The paper of Boulebd [42] describes a theoretical comparative study of the antiradical properties of six aromatic compounds, namely eugenol, safrole, myristicin, carvacrol, cinnamaldehyde, and isoeugenol. Three main antiradical mechanisms—hydrogen atom transfer, single electron transfer‐proton transfer, and sequential proton loss‐electron transfer—have also been investigated. In addition, the Gibbs free energies related to the reactions of the studied compounds with two reactive oxidant species (HO^•^ and HOO^•^) have been computed. It was found that isoeugenol scavenges free radicals through a CH bond and an OH bond. In the gas and benzene phases, all the studied compounds prefer to undergo hydrogen atom transfer mechanism, while in water, sequential proton loss electron transfer is more favored for isoeugenol. The antiradical potential of eugenol, safrole, myristicin, and isoeugenol is similar and superior to that of carvacrol and cinnamaldehyde [42].

Despite isoeugenol’s proven antioxidant activity, none of these articles demonstrated its correlation with NRF2 activation. Only recently, Endo et al. [43] constructed a zebrafish‐based assay system to analyze the in vivo antioxidant activity of isoeugenol. Using hydrogen peroxide and arsenite as oxidative stressors, they conducted a genetic analysis of NRF2 dependency with an Nrf2‐mutant zebrafish line. Their findings demonstrated that isoeugenol significantly reduced arsenite toxicity in an NRF2‐dependent manner and was the first study linking the antioxidant activity of isoeugenol to this signaling pathway [43].

### 3.2. Anti‐Inflammatory Properties

The transcription nuclear factor κB (NF‐κB) is a signaling molecule that, when activated, is responsible for the over‐expression of pro‐inflammatory cytokines, such as tumor necrosis factor α (TNF‐α), interleukin 6 (IL‐6) and IL‐1β, cyclooxygenase‐2 (COX‐2) and inducible nitric oxide synthase (iNOS) [44, 45]. Isoeugenol demonstrates the capacity to prevent the nuclear translocation of NF‐κB, resulting in the suppression of COX‐2 and iNOS expression and the subsequent production of nitric oxide (NO). The down‐regulation of NF‐κB enables the inhibition of cellular damage induced by inflammation [46, 47] and numerous studies suggest its role in the prevention and treatment of various pathologies linked to inflammatory and antioxidant pathways, such as arthritis, inflammatory bowel diseases, and neuroinflammation in neurodegenerative diseases [48, 49]. In accordance, Choi and colleagues investigated the anti‐inflammatory properties of isoeugenol on the transcription factor NF‐κB in the RAW 264.7 mouse macrophage cell line stimulated with lipopolysaccharide (LPS). Their findings demonstrated a notable reduction in iNOS protein levels and nitric oxide synthase type 2 (Nos2) mRNA expression. The study elucidated that the downregulation of iNOS protein, leading to a decrease in NO synthesis, resulted from isoeugenol’s ability to block signaling upstream of the NF‐κB pathway. Additionally, isoeugenol decreased its DNA binding activity due to decreased nuclear translocation of p65, one of the proteins forming the NF‐κB complex [46]. The previous investigation conducted by Li and colleagues equally assessed the impact of isoeugenol on NO production. Isoeugenol inhibited LPS‐dependent production of NO, which was due to the inhibition of iNOS synthesis. Furthermore, LPS‐dependent expression of the COX‐2 protein was markedly inhibited by isoeugenol [47].

Kaur and Sultana investigated the efficacy of isoeugenol in treating adjuvant‐induced arthritis in a murine model. Arthritis, characterized by chronic inflammation, prompted the examination of isoeugenol’s impact on cartilage and bone damage, as well as the modulation of key pro‐inflammatory mediators implicated in the pathophysiology of the condition [49]. These findings demonstrated the isoeugenol’s capacity to inhibit the release of pro‐inflammatory mediators and attenuate levels of pro‐inflammatory enzymes. Accordingly, isoeugenol mitigates arthritis progression by suppressing inflammation and leukocyte infiltration, while preserving cartilage and bone integrity. Remarkably, these effects are dose‐dependent. Unlike many conventional anti‐inflammatory drugs, isoeugenol stands out for its lack of adverse effects on gastric mucosal tissues [49]. The investigation conducted by Zarlaha and colleagues was based on molecular docking assays. The authors revealed the potential of isoeugenol to inhibit the COX‐2 and lipoxygenase‐5 (LOX‐5) enzymes, consequently attenuating the inflammatory response [50]. As previously noted, in the aforementioned articles, the inhibition of the inflammatory response triggered by isoeugenol was never correlated with the activation of NRF2.

### 3.3. The Role of Isoeugenol in Diabetes Mellitus

An increasing body of scientific evidence indicates that NRF2 plays a key role in regulating redox balance, protein homeostasis, and metabolic processes, thereby modulating the progression of diabetes. Experimental studies have underscored the importance of proper NRF2 function in preventing the onset of diabetic complications, highlighting its potential as a therapeutic target for managing the disease [2, 4, 51]. Interestingly, most scientific studies on isoeugenol focus on its effects on diabetes, exploring its potential therapeutic benefits in disease management; however, as previously noted for the other pharmacological activities, these studies have not yet established a direct correlation with NRF2 activation. The research of Rauscher and colleagues has focused on the effects of isoeugenol on glutathione balance and the activity of SOD, CAT, GPX, and glutathione reductase in the liver, heart, brain, and kidney. The study aimed to evaluate the potential of isoeugenol therapy in reversing diabetic oxidative damage. Treatment with isoeugenol reversed diabetic effects on hepatic glutathione peroxidase activity and oxidized glutathione concentration in the brain. Treatment with the lipophilic compound isoeugenol also decreased lipid peroxidation in both the liver and heart of healthy animals and decreased hepatic oxidized glutathione content in both healthy and diabetic rats. Some effects of isoeugenol treatment in diabetic rats, such as decreased activity of hepatic superoxide dismutase and glutathione reductase, were unrelated to the oxidative stress associated with diabetes [52].

A study conducted by Kim and colleagues sought to elucidate the hypoglycemic effects of isoeugenol in rat skeletal muscle cells, particularly in myotubes. In this context, isoeugenol triggered the activation of AMP‐activated protein kinase (AMPK), an enzyme responsible for facilitating glucose uptake in the absence of insulin, and elevated the levels of intracellular calcium, thereby enhancing glucose absorption [53]. In more detail, the authors identified an increase in the phosphorylation of protein kinase C‐α (PKCα) induced by calcium homeostasis alterations, an increase in the expression of glucose transporter type 4 (GLUT4), and its translocation to the plasma membrane. Interestingly, AMPK functions as a metabolic sensor, responding to a decrease in the cell’s energy content. Once activated, AMPK phosphorylates regulatory enzymes in metabolic pathways and indirectly influences gene expression by inhibiting ATP‐consuming pathways and promoting those that generate ATP. Consequently, the activation of this enzyme by isoeugenol leads to enhanced glucose uptake, thereby promoting a hypoglycemic effect [53]. The research carried out by Topal and colleagues demonstrated, through the determination of the inhibition constant (*K*_i_) and the average inhibitory concentration (IC_50_), that isoeugenol exhibits inhibitory effects against certain metabolic enzymes, including α‐glycosidase and α‐amylase. These enzymes play a fundamental role in the digestion of glycogen and starch, being responsible for the hydrolysis of polysaccharides. Inhibiting these enzymes contributes to the postponement of postprandial glucose absorption, influencing postprandial glucose levels and, consequently, reducing hyperglycemia [54]. In the study conducted by Alharthy and colleagues, the effects of isoeugenol and eugenol and its combination were evaluated on weight changes, blood glucose levels, food and water consumption, antioxidant parameters, TNF‐α and nerve growth factor levels, histology of sciatic nerve, hyperalgesia, and allodynia in streptozotocin‐induced neuropathic diabetic rats where polyol pathway hyperactivity resulting from chronic hyperglycaemia is the main underlying pathological mechanism. The authors concluded that the protection against oxidative stress in peripheral nerve cells was conferred with greater efficacy in the combined treatment of isoeugenol and eugenol. The authors also observed a significant decrease in lipid peroxidation and an increase in SOD, CAT, and GSH [55]. Alharthy and colleagues also documented alterations in the number of macrophages and the levels of pro‐inflammatory cytokines, specifically TNF‐α, in the group submitted to treatment. Furthermore, isoeugenol and eugenol increased nerve growth factor levels, which play a crucial role in the positive regulation of the sympathetic and sensory nervous system. These changes were attributed to the anti‐inflammatory properties and the ability of both molecules to alleviate oxidative stress [55]. In the year before, Martiz et al. [56] investigated the therapeutical potential of *Ocimum tenuiflorum* at multiple stages of diabetes, using target‐based computational techniques for α‐glucosidase, α‐amylase, aldose reductase, and glycation. It aimed to elucidate the mechanism by which phytochemicals of *O. tenuiflorum*, such as isoeugenol, treat diabetes mellitus using concepts of drug‐likeness and pharmacokinetics, molecular docking simulations, molecular dynamics simulations, and binding free energy studies. Isoeugenol was found to inhibit all the target enzymes, with a higher binding efficiency than standard drugs. Furthermore, molecular dynamic experiments revealed that isoeugenol was more stable in the binding pockets than the standard drugs used. The authors emphasized the superior binding efficiency and stability of isoeugenol, suggesting its potential as a widely employed lead inhibitor of α‐glucosidase, α‐amylase, aldose reductase, and glycation. This highlights its efficacy as a treatment for hyperglycemia and diabetes mellitus [57].

Recently, Hao and colleagues conducted a study comparing the efficacy of an isoeugenol‐derived compound, eugenosedin‐A, with glibenclamide and pioglitazone in protecting against diabetic‐induced cardiovascular dysfunction in spontaneously hypertensive rats. All the drugs significantly ameliorated changes in body weight, cardiac weight, biochemical parameters, cardiovascular disorders, and inflammation. Therefore, eugenosedin‐A, similar to glibenclamide and pioglitazone, may effectively control diabetes mellitus and the associated cardiovascular dysfunction [58]. Very recently, the effects of isoeugenol on adipogenesis were elucidated. Adipogenesis plays a crucial role in obesity, which arises from an imbalance between energy intake and expenditure and leads to metabolic diseases, including cardiovascular diseases, diabetes, and liver diseases. Isoeugenol inhibits adipogenesis in 3T3‐L1 cells by blocking lipid accumulation and reducing the expression of adipocyte‐related factors such as PPARγ, C/EBPα, and FABP4. It arrests preadipocytes in the G0/G1 phase and inhibits adipocyte differentiation suggesting its potential as a therapeutic agent for obesity and associated metabolic diseases, including diabetes [59].

Overall, research has shown that isoeugenol can positively influence glucose metabolism, enhance insulin sensitivity, and reduce oxidative stress and inflammation, as well as associated cardiovascular complications and obesity, which are key factors in the progression of diabetes. However, none of these studies correlated the therapeutic effects of isoeugenol with its intrinsic capacity for NRF2 activation. While this link is highly plausible, it still needs to be confirmed.

### 3.4. The Role of Isoeugenol in Alzheimer’s Disease (AD)

From a pathophysiological perspective, AD is characterized by the loss of homeostatic functions that control redox and energy metabolism, neuroinflammation, and proteostasis. NRF2 is a master controller of these functions and, in the past decades, recent reports have shown that its overall activity is compromised in AD [6, 8]. Thus, NRF2 is an attractive molecular target for therapeutic research in AD. Using this rationale, Silva et al. [48] investigated whether DMF and other unexplored electrophilic skin allergens could have a preventive and/or therapeutic role in AD. In a first approach, an extensive physical library of allergens was screened in terms of hydrophobicity, drug‐likeness properties and toxicity prediction to select a set of compounds able to be absorbed at the gastrointestinal tract, to cross the blood–brain barrier and to exert low to moderate toxicity. Besides DMF two drug‐like electrophiles unexplored in the context of neurodegenerative diseases, 1,4‐phenylenediamine (PPD) and methyl heptine carbonate (MHC) that, similarly to DMF, react with cysteine residues, were screened. The antioxidant and anti‐inflammatory properties of these molecules were studied in a mouse neuronal cell line (N2a), wild‐type (N2a‐wt) or overexpressing human wild‐type APP (N2a‐APPwt), as an AD neuronal model, and in a mouse microglia cell line (BV‐2) stimulated with LPS, as an inflammation model. The results achieved demonstrated that DMF, PPD, and MHC increased *Hmox1* gene expression and HO‐1 protein levels in N2a‐APPwt cells suggesting NRF2‐dependent antioxidant activity. All the chemicals studied showed anti‐inflammatory activity by decreasing iNOS protein expression in LPS‐exposed microglia. More recently, and using the same rationale, the same group explored the skin allergen isoeugenol, in in vitro and in vivo models of AD [60]. Accordingly, the antioxidant and anti‐inflammatory properties of isoeugenol were studied in vitro using an AD neuronal model (N2a‐wt and human APP with Swedish mutation overexpressing cells ‐ N2a‐APPswe) and a neuroinflammation model (BV‐2 exposed to LPS), respectively. In vivo studies were also conducted in APPswe/PSEN1dE9 double transgenic mice (APP/PS1) and their wild‐type (WT) counterparts at early (6‐month‐old (mo) mice) and late (11 mo) AD stages. Isoeugenol was administered, for 1 month, intranasally. The results showed that isoeugenol activated the NRF2 pathway, creating a more favorable antioxidant, anti‐inflammatory, metabolic, and proteostasis environment that can limit disease progression. The activation of NRF2 by isoeugenol is likely evoked by the covalent modification of cysteine residues in KEAP1. However, the involvement of the PI3K/Akt/GSK pathway may also play a role, as evidenced by increased AKT activation and GSK3β inactivation observed in N2a‐APPswe cells treated with isoeugenol. Pharmacokinetic studies have demonstrated that isoeugenol is absorbed and distributed in the plasma and brain after intranasal administration. Moreover, histopathological analysis showed minor differences between the untreated and treated animals, suggesting the safety of isoeugenol administration. Isoeugenol decreased the levels of Aβ in vitro and in the brains of APP/PS1 transgenic mice at 6 and 11 months of age, as well as beta‐secretase 1 (*Bace1*) gene expression, and increased heme oxygenase 1 (*Hmox1*) in the hippocampus. Importantly, Isoeugenol‐improved cognition, particularly at 11 months. Overall, the results confirmed that isoeugenol has a neuroprotective role in both in vitro and in vivo models of AD, highlighting its therapeutic potential for the treatment of this disease. Importantly, this study was the second to correlate the therapeutic properties of isoeugenol with its capacity to activate NRF2.

Another study by Topal et al. [54] demonstrated that isoeugenol inhibits the acetylcholinesterase enzyme, a key molecular target overexpressed in AD. Notably, current AD treatments such as galantamine, rivastigmine, and donepezil are all acetylcholinesterase inhibitors, highlighting the potential of isoeugenol in this therapeutic area. Scientific evidence has established a biomolecular link between neurodegenerative diseases, particularly AD, and Type 2 Diabetes (T2D). This connection stresses an association between cognitive function impairments and hyperglycaemia [61]. AD and T2DM share several pathological mechanisms, including the downregulation of glucose transporters, impaired insulin signaling pathways, neuroinflammation, and mitochondrial dysfunction [62]. These overlapping mechanisms suggest a correlation between the two conditions, often referred to as brain‐specific T2D or type 3 diabetes [63]. Using network pharmacology tools, Martiz and colleagues reported the virtual screening of isoeugenol as an inhibitor of glyceraldehyde 3‐phosphate dehydrogenase (GAPDH), a common target for both T2D and AD. Isoeugenol was identified as the most effective GAPDH inhibitor among the molecules present in *Ocimum tenuiflorum*. Computational analyses, including molecular docking, molecular dynamics simulations, and binding free energy calculations, confirmed the binding interactions and stability of isoeugenol with GAPDH. These findings suggest that isoeugenol could be a promising GAPDH inhibitor, meriting further in vitro and in vivo studies for T2D‐linked AD [64].

## 4. Conclusions

**Figure 6:**
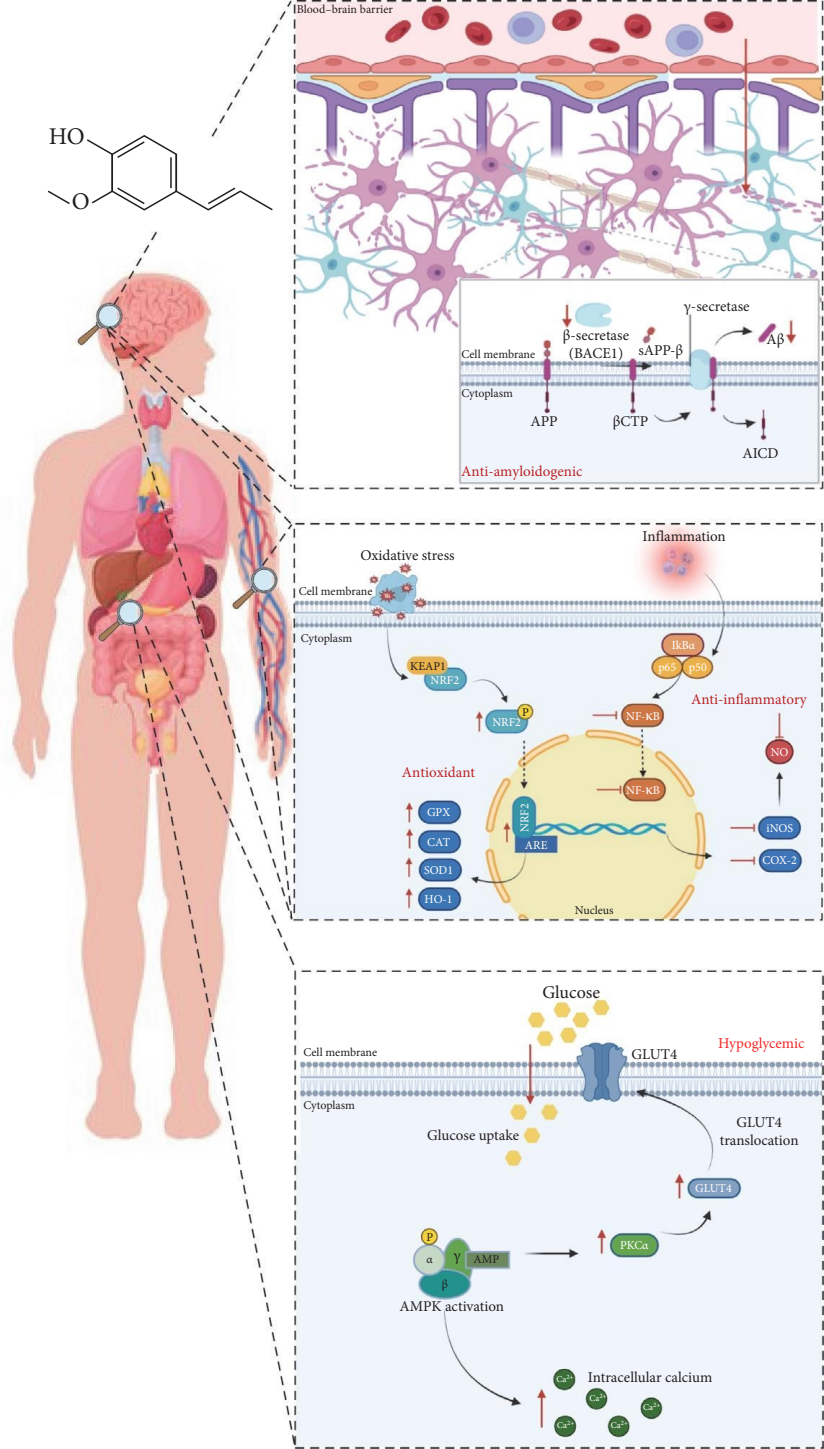
Mechanism of action of isoeugenol. Isoeugenol reacts with the cysteine residues of KEAP1, causing KEAP1 to dissociate from NRF2 leading to nuclear translocation of NRF2. NRF2 then binds to the ARE and drives the expression of antioxidant target genes, such as *HMOX1*, *SOD1*, *CAT*, and *GPX*. Isoeugenol inhibits nuclear translocation of p65, leading to the inhibition of NF‐κB, thus decreasing the production of pro‐inflammatory cytokines. Additionally, isoeugenol triggers the activation of AMPK, facilitating glucose uptake in the absence of insulin, and increasing the levels of intracellular calcium, thereby enhancing glucose absorption. Furthermore, isoeugenol crosses the blood–brain barrier, decreases neuroinflammation, and exhibits activity against the amyloidogenic cascade by decreasing the β‐secretase activity and the Aβ peptide levels.

The cellular balance between ROS and antioxidants is crucial for maintaining homeostasis. Disruption of this balance, due to impaired defense mechanisms or increased free radical production, leads to oxidative stress, causing significant damage to cellular components and ultimately resulting in tissue damage and cell death. The transcription factor NRF2 plays a critical role in responding to oxidative stress by regulating a network of protective genes. The NRF2–KEAP1 signaling pathway, which evolved to mitigate the toxicity of ROS, has been fundamental for survival across various environmental conditions and has emerged as a promising therapeutic strategy for diseases characterized by oxidative stress and inflammation. Low molecular weight skin allergens are well‐known activators of NRF2. These allergens, traditionally associated with toxic effects, may offer therapeutic benefits when harnessed correctly. Particularly, isoeugenol, a phenylpropanoid found in essential oils, has shown promise in various studies, likely due to its ability to activate NRF2. However, few articles have correlated its therapeutic properties—namely, antioxidant, anti‐inflammatory, neuroprotective, and modulator of metabolic disorders such as diabetes mellitus (Figure 6)—with its intrinsic capacity to activate NRF2. While this link is highly plausible, further research is needed to confirm its role in the therapeutic effects attributed to isoeugenol. Based on all the scientific information available, it can be hypothesized that the activation of NRF2 by isoeugenol likely occurs through three primary mechanisms: the covalent modification of cysteine residues in KEAP1, increased AKT activation and GSK3β inactivation, and glutathione depletion leading to ROS generation, which serves as the golden standard activator of NRF2. This activation not only counterbalances ROS but also triggers protective responses, such as anti‐inflammatory effects, inhibition of ferroptosis, and enhanced autophagy, all of which contribute to cellular survival and tissue homeostasis.

In conclusion, isoeugenol and similar compounds offer a promising new avenue for therapeutic development, particularly in conditions related to oxidative stress and inflammation. However, further investigation is needed to better understand their target specificity, pharmacodynamic properties, efficacy, and safety. In addition to the reported beneficial activities, isoeugenol exhibits a suboptimal pharmacokinetic profile, which likely limits its therapeutic potential. To fully realize its benefits, targeted pharmaceutical modifications would be necessary to enhance its pharmacokinetic properties, ensuring improved absorption, distribution, and sustained efficacy for clinical use. Further research on these small molecules could lead to novel strategies for addressing chronic diseases by harnessing the protective effects of Nrf2 activation. This review highlights isoeugenol’s potential as a multi‐disease therapeutic agent and advocates for its inclusion in drug discovery programs focused on its Nrf2‐mediated protective effects.

## Disclosure

Views and opinions expressed are however those of the authors only and do not necessarily reflect those of the European Union or European Health and Digital Executive Agency (HADEA), under the powers delegated by the European Commission.

## Conflicts of Interest

The authors declare no conflicts of interest.

## Author Contributions

Ana Silva and Sónia Silva contributed equally to this work and should be listed as co‐first authors.

## Funding

This work was financed by COMPETE 2020–Operational Programme for Competitiveness and Internationalisation and Portuguese national funds via FCT – Fundação para a Ciência e a Tecnologia, under projects POCI‐01‐0145‐FEDER‐029369 and UIDB/04539/2020, UIDP/04539/2020 and LA/P/0058/2020 and also supported by the COST Action CA20121, Bench to bedside transition for pharmacological regulation of NRF2 in noncommunicable diseases (BenBedPhar) and European Union, Grant Agreement Number 101080329 (Project PAS GRAS).

## Data Availability

The datasets analyzed during the current study are available from the corresponding author upon request.
